# Rare Case Studies of Bilateral and Symmetric Sacroiliac Disease

**DOI:** 10.1155/2024/8893089

**Published:** 2024-03-07

**Authors:** Stephen Soloway, Alyxandra M. Soloway, Tyler G. Chin, Timothy Lieske

**Affiliations:** ^1^Rheumatology, Department of Internal Medicine, Inspira Health Network, Vineland, USA; ^2^Internal Medicine Resident PGY3, Inspira Health Network; PGY4, Tampa General Hospital Rheumatology Fellow, Tampa, FL, USA; ^3^Ursinus College, Collegeville, PA, USA; ^4^Arthritis and Rheumatology Associates, Vineland, NJ, USA

## Abstract

**Introduction:**

Inflammatory sacroiliitis is common in rheumatology practice. Spondyloarthritis is often underdiagnosed due to the lack of proper evaluation of the sacroiliac joints (SIJs), clinically and radiographically. If SIJ is inflamed or arthritic, the arthritic said patient typically has spondyloarthritis, in the absence of infections or crystal arthritis. Sacroiliitis, in particular, when diagnosed between 12 and 45 years of age, is indicative of spondyloarthritis. People are often misdiagnosed and mislabeled as fibromyalgia because their serologies are negative. Our goal is to point out the importance of proper evaluation, diagnosis, and importance of inflammatory SIJ disease and conditions that involve SIJ inflammation.

**Cases:**

We present three rare conditions presenting with bilateral and symmetric SIJ disease, none of which is ankylosing spondylitis, Crohn's colitis, ulcerative colitis, psoriatic arthritis, and reactive arthritis (Reiter syndrome); there are reports of concurrent SIJ disease in rheumatoid arthritis and SLE.

**Conclusion:**

The authors believe that SIJ disease is overlooked, is underdiagnosed, and can lead to incorrect treatment. We suggest a greater focus on SIJ imaging in the diagnosis and treatment of unexplained illnesses associated with low back pain, morning stiffness, or unexplained buttock pain. Providers should review their own SIJ films. The meaning of SIJ widening, cortical irregularity, spurs, and the significance of the anterior inferior SI joints, bone marrow edema, and fusion (namely, the natural history of sacroiliac pathophysiology).

## 1. Introduction

Inflammatory sacroiliitis is quite common in the practice of rheumatology. The reported literature indicates that 2% of the population is affected by inflammatory sacroiliitis, while gouty arthritis, the most common inflammatory arthritis, occurs in 5% of the population [1, 2]. However, we have strong opinions similar to that of Muhammad Asad Khan (personal communication) that these numbers are falsely suppressed due to lack of skills in reading sacroiliac films. Spondyloarthritis is commonly underdiagnosed due to the lack of proper evaluation of the sacroiliac joints (SIJs). This is due to the fact that they are underread on diagnostic imaging by radiologists or rheumatologists. Clinicians call it rheumatoid arthritis or seronegative rheumatoid arthritis to begin biologic treatment without looking at SIJ. Once the SIJ is deemed abnormal, that person really has spondyloarthritis. Doctors use the term “working diagnosis” of ankylosing spondylitis or psoriatic arthritis or occasionally inflammatory bowel arthritis. Sacroiliitis, particularly in the younger population between 12 and 45 years, is indicative of spondyloarthritis. Classic spondyloarthritis includes ankylosing spondylitis, psoriatic arthritis, reactive arthritis, and inflammatory bowel arthritis (Crohn's colitis and ulcerative colitis). Diagnoses of exclusion include septic joint, as seen in IV drug use, and other pyogenic infections, rarely crystals [3, 4].

People are often misdiagnosed and mislabeled because their serologies are negative or are often incorrectly diagnosed as fibromyalgia. This article is important for exemplifying the overlooked diagnosis of sacroiliitis. The importance of accurate SIJ X-ray reading between a skilled rheumatologist and a musculoskeletal radiologist is imperative but overlooked. Traditionally, bilateral and symmetric SIJ disease is seen in ankylosing spondylitis, Crohn's colitis, or ulcerative colitis while unilateral and asymmetric disease is seen in psoriatic arthritis and reactive arthritis [5–8].

Less common diseases may present with SIJ involvement; although easy to diagnose, it remains a challenge to obtain an early evaluation of SIJ and there appears to be a lack of rheumatology involvement when needed most. We chronicle three less common conditions seen in rheumatology practice presenting with SIJ disease. Behcet's disease (a large vessel vasculopathy with predilection for recurrent mucocutaneous ulcer and pulmonary artery aneurysm), and celiac disease, (occurrence can be 20% at presentation), a small bowel malabsorption with anemia, bloating, gluten sensitivity and HLA DQ2 and HLA DQ8, and VKH (Vogt–Koyanagi–Harada syndrome: granulomatous panuveitis and sensorineural hearing loss), with Whipple's disease, and sarcoidosiss. It is well established that spondyloarthropathies have many common clinical findings including papulosquamous skin lesions also known as psoriasis, circinate balanitis, dermatitis herpetiformis, HS, ocular disease most commonly uveitis (anterior, posterior, and pan), enthesitis, which occurs in the Achilles, plantar fascia, and (costochondral) rib pain. SIJ disease and posterior uveitis may support the diagnosis of sarcoidosis of Behcet's and Whipple's disease. VKH and granulomatous posterior uveitis are not typically seen with SIJ disease; however, we describe the onset of VKH (the first reported case), Behcet's, and celiac disease, all of which present with sacroiliitis. We believe this is under-reported in published data. An IRB exemption has been received.

## 2. Cases

Patient one is a 60-year-old Vietnamese man who presented with mouth and genital ulcers, a long history of pulmonary artery aneurysm and erythema nodosum, and low back and buttock pain with 90 minutes of stiffness in the morning. He was lost to follow-up and returned after 10 years. Upon presentation, the patient had stiffness in the morning for more than an hour and plain film X-rays revealed bilateral sclerosis of the anterior inferior portion of the iliac portion of the SIJ bilaterally. At the same time, the patient had panuveitis. He was referred to a retinal specialist who diagnosed panuveitis and started corticosteroid eye drops; after initial encounter with this patient based on the history of pulmonary artery aneurysm and nongranulomatous panuveitis with bilateral SIJ disease, chest imaging with contrast-enhanced computed tomography revealed no interstitial disease or adenopathy; this patient was labeled as having Behcet's syndrome and was treated with infliximab 5 mg/kg and remitted.

Patient 2, a 27-year-old Hispanic woman, presented with a prior diagnosis of VKH. The patient was presented to rheumatology department for treatment with TNF inhibitors (Humira and etanercept) for noninfectious granulomatous panuveitis. The patient did not show deafness or other stigmata of VKH; however, in evaluation at rheumatology department, this was a little unusual, SIJ films were performed, and plain film imaging showed bilateral SIJ disease (sclerosis along the anterior inferior iliac margins bilaterally confirmed with hyperintense MRI lesions in the same area representing edema and erosions).

Two diseases such as Behcet's is noted to occur with sacroiliitis [9] but often overlooked and in fact can present with sacroiliitis and VKH is not known to present with sacroiliitis, nor does sacroiliitis occur in the context of VKH (inflammatory noninfectious granulomatous panuveitis). They were tested negative for ANA, ANCA, MPO, PR3 RF, HLA B27, QuantiFERON Gold, RPR, ACE, CXR, HLA-B51, HLA A29, IgA-TTG, and (Histoplasmosis, Blastomycosis, and Coccidioides). The skin test was negative, and the patient was ergic.

Patient 3 is a 37-year-old white male of North European ancestry with a strong family history of type 1 diabetes who came to the clinic with severe bilateral buttock pain with three hours of morning stiffness; in addition, there was a long history of weakness, arthralgias, and unusual floating stools as stated in the patient's words. He did not have severe abdominal pain or cramping; however, he was tested positive for HLA DQ 2 and HLA DQ 8. His IGA tissue (IgA-TTG) transglutaminase was greater than 100 units/ml. Due to buttock pain, the patient had imaging of SIJ after X-ray, bilateral iliac sclerosis with narrowing, and marrow edema on MRI. Small bowel biopsy showed classic findings of celiac disease; flattening of the duodenal villi on EGD; osteoporosis of the femoral neck; mean lumbar *T* score and *Z* score as −3.0 and −2.9, respectively; dermatitis herpetiformis, mouth ulcers, headaches, fatigue, and 8 gm anemia.

In conclusion, we present three conditions with bilateral symmetric SIJ disease, which are often overlooked; none had ankylosing spondylitis, Crohn's colitis, ulcerative colitis, psoriatic arthritis, reactive arthritis (Reiter syndrome), or sarcoidosis. The authors believe that SIJ disease is overlooked, is underdiagnosed, and can lead to incorrect treatment, while in other parts of the world, it can be overdiagnosed. We suggest a greater focus on SIJ in the diagnosis and treatment of unexplained illnesses associated with low back or buttock pain. We believe that rheumatology is underutilized as many health professionals do not understand the scope of a rheumatologist, and imaging of SIJ when done is often overlooked, underlooked, or misread. Whether underdiagnosed or overdiagnosed, it is important to point out all the findings seen, such as joint space widening, pseudowidening, sclerosis, osteophytes, cysts, or narrow edema. We must agree that “OA,” when described in a teenager, is a pathological finding and cannot be assumed to be normal.

## Data Availability

All data are HIPAA protected and may be made available upon personal request.

## Supplementary Materials

Supplementary Materials Supplementary materials include the Ap Sacroiliac Image #1: bilateral SIJ disease, AP Sacroiliac Image #2: with sclerosis along anterior and inferior iliac margins bilaterally, and AP Sacroiliac Image #3: bilateral iliac sclerosis with narrowing, marrow edema on MRI.
