# Supplementary material for: Rare Case Studies of Bilateral and Symmetric Sacroiliac Disease
Source: Case Rep Rheumatol. 2024 Mar 7;2024:8893089. doi: 10.1155/2024/8893089 (PMC10940025; doi:10.1155/2024/8893089)

Rare Case Studies

Bilateral And Symmetric Sacroiliac Disease

Ap Sacroiliac Image #1 Bilateral SIJ Disease

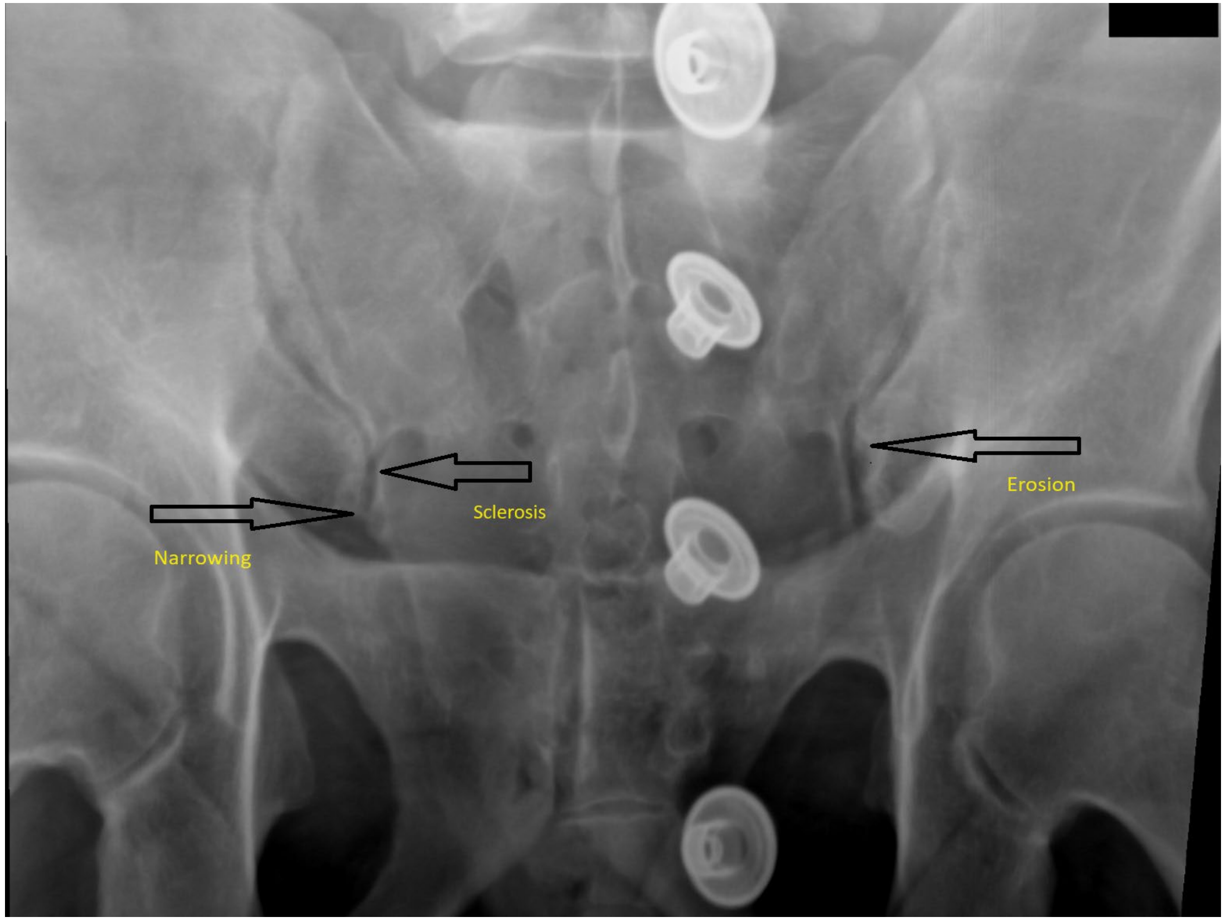

AP Sacroiliac Image #2 with sclerosis along interior, inferior iliac margins bilaterally

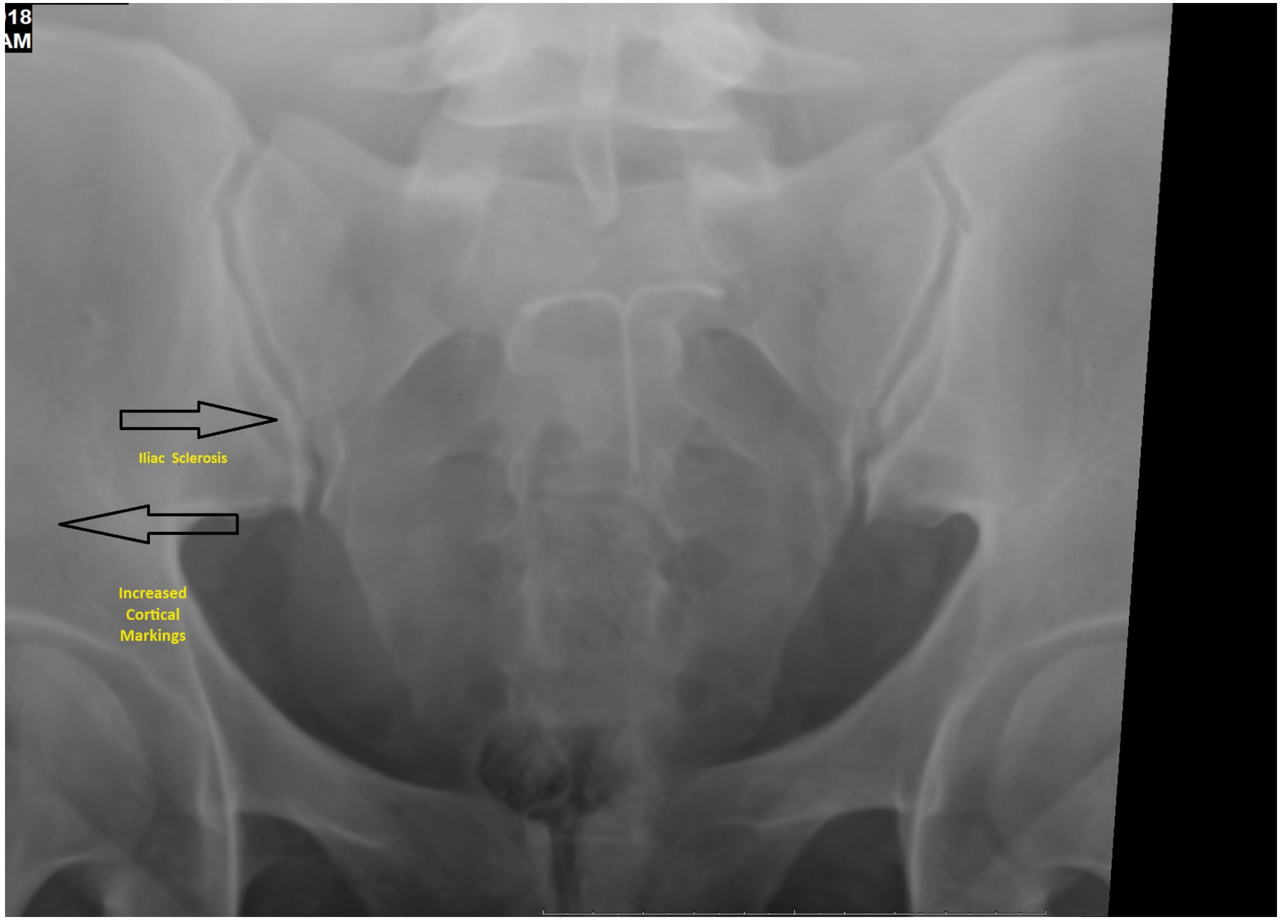

AP Sacroiliac Image #3 –Bilateral Iliac Sclerosis with narrowing, marrow edema on MRI

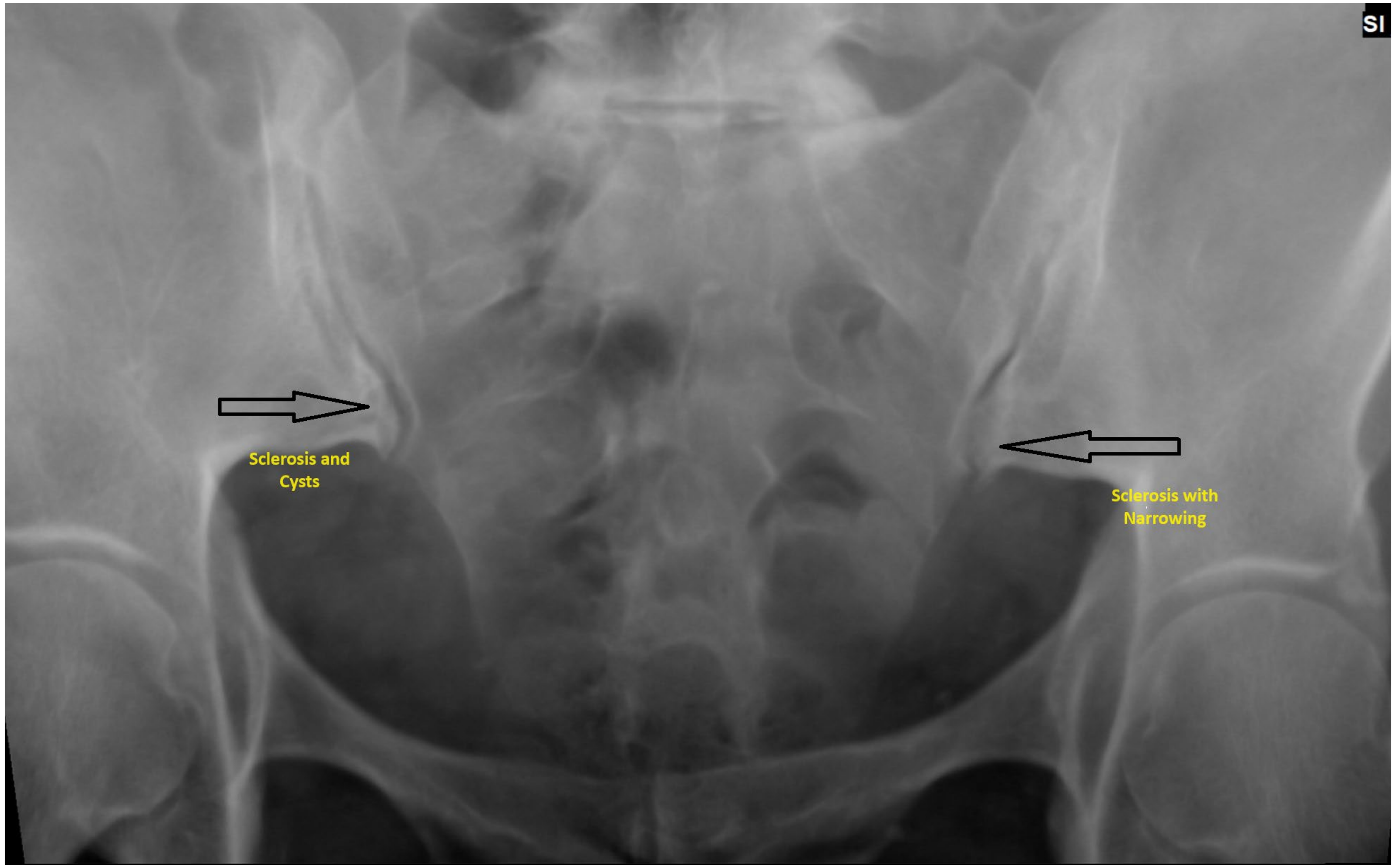

Supplement: Supplementary Materials — include the Ap Sacroiliac Image #1: bilateral SIJ disease, AP Sacroiliac Image #2: with sclerosis along anterior and inferior iliac margins bilaterally, and AP Sacroiliac Image #3: bilateral iliac sclerosis with narrowing, marrow edema on MRI. [file 8893089.f1.pdf]
